# The Multilingual Picture Database

**DOI:** 10.1038/s41597-022-01552-7

**Published:** 2022-07-21

**Authors:** Jon Andoni Duñabeitia, Ana Baciero, Kyriakos Antoniou, Mark Antoniou, Esra Ataman, Cristina Baus, Michal Ben-Shachar, Ozan Can Çağlar, Jan Chromý, Montserrat Comesaña, Maroš Filip, Dušica Filipović Đurđević, Margaret Gillon Dowens, Anna Hatzidaki, Jiří Januška, Zuraini Jusoh, Rama Kanj, Say Young Kim, Bilal Kırkıcı, Alina Leminen, Terje Lohndal, Ngee Thai Yap, Hanna Renvall, Jason Rothman, Phaedra Royle, Mikel Santesteban, Yamila Sevilla, Natalia Slioussar, Awel Vaughan-Evans, Zofia Wodniecka, Stefanie Wulff, Christos Pliatsikas

**Affiliations:** 1 Centro de Investigación Nebrija en Cognición (CINC), Universidad Nebrija, Madrid, Spain; 2 UiT The Arctic University of Norway, Tromsø, Norway; 3 Bournemouth University, Poole, United Kingdom; 4 Department of Rehabilitation Sciences, Cyprus University of Technology, Limassol, Cyprus; 5 Hellenic Open University, Patras, Greece; 6 The MARCS Institute for Brain, Behaviour and Development, Western Sydney University, Penrith, NSW Australia; 7 School of Psychological Sciences and Centre for Reading, Macquarie University, Sydney, Australia; 8 Department of Cognition, Development and Educational Psychology, Universitat de Barcelona, Barcelona, Spain; 9 Department of English Literature and Linguistics and The Gonda Multidisciplinary Brain Research Center, Bar-Ilan University, Ramat-Gan, Israel; 10 Department of Foreign Language Education, Middle East Technical University, Ankara, Turkey; 11 Institute of Czech Language and Theory of Communication, Faculty of Arts, Charles University, Praha, Czech Republic; 12 Research Unit in Human Cognition, CIPsi, School of Psychology, University of Minho, Braga, Portugal; 13 Department of Linguistics, Faculty of Arts, Charles University, Praha, Czech Republic; 14 Laboratory for Experimental Psychology, and Department of Psychology, Faculty of Philosophy, University of Belgrade, Beograd, Serbia; 15 School of Education and English, University of Nottingham Ningbo China, Ningbo, China; 16 School of Philosophy, Department of English Language and Literature, National and Kapodistrian University of Athens, Athens, Greece; 17 Department of Central European Studies, Faculty of Arts, Charles University, Praha, Czech Republic; 18 Malay Language Department, Faculty of Modern Languages and Communication, Universiti Putra Malaysia, Serdang, Malaysia; 19 School of Psychology and Clinical Language Sciences, University of Reading, Reading, UK; 20 Department of English Language and Literature, and Hanyang Institute for Phonetics and Cognitive Sciences of Language, Hanyang University, Seoul, Republic of Korea; 21 C-unit, Laurea University of Applied Sciences, Vantaa, Finland; 22 Cognitive Brain Research Unit, Department of Psychology and Logopedics, University of Helsinki, Helsinki, Finland; 23 Department of Language and Literature, NTNU Norwegian University of Science and Technology, Trondheim, Norway; 24 AcqVA Aurora Center, Institute of Language and Culture, UiT the Arctic University of Norway, Tromsø, Norway; 25 Department of English, Faculty of Modern Languages and Communication, Universiti Putra Malaysia, Serdang, Selangor Malaysia; 26 Department of Neuroscience and Biomedical Engineering, Aalto University, Espoo, Finland; 27 BioMag Laboratory, HUS Diagnostic Center, Helsinki University Hospital, University of Helsinki and Aalto University, Helsinki, Finland; 28 School of Speech-language Pathology and Audiology, University of Montreal, Centre for Research on Brain, Language and Music (CRBLM), and Interdisciplinary Centre for Brain and Learning Research (CIRCA), Montréal, Québec Canada; 29 The Bilingual Mind Research Group, Department of Linguistics and Basque Studies, University of the Basque Country UPV/EHU, Vitoria-Gasteiz, Spain; 30 Instituto de Lingüística, Facultad de Filosofía y Letras, Universidad de Buenos Aires, Consejo Nacional de Investigaciones Científicas y Técnicas (Conicet), Buenos Aires, Argentina; 31 School of Linguistics, Higher School of Economics, Moscow, Russia; 32 Saint Petersburg State University, Saint Petersburg, Russia; 33 School of Human and Behavioural Sciences, Prifysgol Bangor, Bangor, Wales UK; 34 Institute of Psychology, Jagiellonian University, Krakow, Poland; 35 University of Florida, Gainesville, Florida USA

**Keywords:** Human behaviour, Human behaviour

## Abstract

The growing interdisciplinary research field of psycholinguistics is in constant need of new and up-to-date tools which will allow researchers to answer complex questions, but also expand on languages other than English, which dominates the field. One type of such tools are picture datasets which provide naming norms for everyday objects. However, existing databases tend to be small in terms of the number of items they include, and have also been normed in a limited number of languages, despite the recent boom in multilingualism research. In this paper we present the Multilingual Picture (Multipic) database, containing naming norms and familiarity scores for 500 coloured pictures, in thirty-two languages or language varieties from around the world. The data was validated with standard methods that have been used for existing picture datasets. This is the first dataset to provide naming norms, and translation equivalents, for such a variety of languages; as such, it will be of particular value to psycholinguists and other interested researchers. The dataset has been made freely available.

## Abstract (summary)

**Table:** 

Measurement(s)	Picture naming norms • Picture familiarity ratings
Technology Type(s)	Online experiment

## Background & Summary

Research on the wide and multidisciplinary area of language (e.g., perception, production, processing, acquisition, learning, disorders, and multilingualism, among others) frequently uses pictures of objects as stimuli for different paradigms such as naming or classification tasks. Importantly, experimenters need to have access to normative data on diverse properties of the pictures (e.g., naming agreement, familiarity, or complexity) to be able to compare and generalise their results across studies. Crucially, in a world in which multilingualism is the norm— it has been estimated that more than half of the world’s population speaks two or more languages^1,2^—it is essential for researchers to be able to access such normative information of experimental items for different languages.

Snodgrass and Vanderwart^3^ created the first normalised picture dataset for the American English language, which has been adapted to other languages in order to conduct cross-linguistic research (e.g., British English^4^; Chinese^5^; Croatian^6^; Dutch^7^; French^8^; Argentinian Spanish^9^; Italian^10^; Japanese^11^; Spanish^12^). However, these datasets involve black and white line-drawings, which have been shown to generate weaker recognition than coloured pictures^13,14^. Considering these findings, researchers have developed coloured image datasets, also in different languages (e.g., English^15^; French^13^; Italian^16^; Russian^17^; Modern Greek^18^; Turkish^19^; Spanish^20^).

Despite all these efforts to develop standardised and open datasets of pictures and their properties in different languages, there are still some limitations. First, these datasets typically only include around 300 images (except for English^15^ and Canadian French^15^), which greatly restrict experimental designs. Second, these datasets were created independently of one another, and hence, they were normalised using different protocols. To overcome these limitations, we have created a database of 500 coloured pictures of concrete objects for 32 different languages or language varieties (i.e., American English, Australian English, Basque, Belgium Dutch, British English, Catalan, Cypriot Greek, Czech, Finnish, French, German, Greek, Hebrew, Hungarian, Italian, Korean, Lebanese Arabic, Malay, Malaysian English, Mandarin Chinese, Netherlands Dutch, Norwegian, Polish, Portuguese, Quebec French, Rioplatense Spanish, Russian, Serbian, Slovak, Spanish, Turkish, Welsh) using the same procedure for data collection and preprocessing. To this end, we developed a procedure similar to that reported in Duñabeitia *et al*.^21^ who created the initial dataset, which included 750 coloured images standardised for six commonly spoken European languages (i.e., British English, Dutch, French, German, Italian, and Spanish).

This Data Descriptor describes, in a comprehensive manner, the experimental method, the preprocessing protocol, and the structure of the data. Our aim is to make this database freely available to all researchers so that they can conduct empirical studies in any language. This is especially interesting for researchers concerned with any multilingual issue, since it offers them the opportunity to design studies for which the properties of the materials have been tested in a parallel manner for all the languages in their study. The datafile containing the whole dataset has been stored in a public repository^22^, and we encourage any researcher to use it for their studies.

## Methods

We selected 500 coloured pictures with the highest name agreement across languages from a set of 750 pictures created by Duñabeitia *et al*.^21^. These pictures were in PNG format with a resolution of 300 × 300 pixels at 96 dpi and they have been stored in the public repository in a compressed folder for the convenience of readers and potential users. Additionally, given that some users may want to opt for different versions of the PNG pictures^13,14^, the same public repository includes a folder containing black and white and grey scale versions of the same drawings.

The same experimental software was used across sites. To this end, a custom program was generated using Gorilla Experiment Builder^23^ and replicated across languages with exactly the same instructions to ensure homogeneity in the protocols. Participants were told that they would see a series of images, and that they should type in the name of the entity represented in each picture. Each of the pictures was presented individually in the centre of the display of a computer or tablet. Participants were asked to make sure they spelled the word correctly, and try not to use more than one word per concept. If they did not know the name of the element depicted, they could indicate this by typing “?”, and this would then be considered as an “I don’t know” response (see below). After typing the name, they were asked to indicate their self-perceived familiarity with the concept, using a 100-point scale slider (with the lowest value indicating “not familiar at all” and the highest value representing “very familiar”). Participants were asked to use the whole scale during the experiment and avoid using only the extreme values. In order to get used to the procedure, they completed two practice trials before starting the experiment. The entire experiment lasted about one hour, and breaks were inserted during the test at every 50 trials.

The data were collected during 2020 and 2021 in the context of a large-scale crowdsourcing study. Ethical approval for conducting the general study was obtained from the Ethics Committee of Universidad Nebrija (approval code JADL02102019), and from the participating institutions that required individual extensions or ethics approval from their local ethics boards. The data preprocessing procedure included checking the answers for spelling errors by native speakers of each language and merging variants of the same response, following the procedure described in Duñabeitia *et al*.^21^.

These datasets were then combined with the data for the 500 pictures extracted from the original study^21^ regarding Belgium Dutch, British English, French, German, Italian, Netherlands Dutch, and Spanish. In the original study, speakers of different languages were also asked to rate following a 1-to-5 scale the visual complexity of the drawings, and results showed a very high cross-linguistic correlations (with r-values larger than 0.90). For this reason, and considering that those visual complexity scores are readily available from the original study can be applied to the new set of languages reported here, in the current multi-centre study we decided to focus on familiarity as a different dimension that could vary across cultures. At this regard, it is worth noting that even if the original set of languages reported in Duñabeitia *et al*.^21^ did not include familiarity ratings, these could be easily obtained from published databases (e.g., British English^24^, Dutch^25^, French^26^, German^27^, Italian^10^, Spanish^28^). Together, data from a total of 2,573 participants are reported. See Supplementary Table for a full description of the dataset.

## Data Records

The dataset resulting from the online testing is freely available in CSV and XLSX formats^22^. Each row in the file represents the aggregated data for one specific item across all participants who completed the test in each language, and each column represents a variable of interest. The column labelled **Language** includes a string referring to the specific language or variety out of the 32 tested to which the data refers (American English, Australian English, Basque, Belgium Dutch, British English, Catalan, Cypriot Greek, Czech, Finnish, French (standard), German, Greek (standard), Hebrew, Hungarian, Italian, Korean, Lebanese Arabic, Malay, Malaysian English, Mandarin Chinese, Netherlands Dutch, Norwegian, Polish, Portuguese, Quebec French, Rioplatense Spanish, Russian, Serbian, Slovak, Spanish, Turkish, or Welsh). The column labelled **Code** includes a number between 1 and 747 corresponding to the picture to which the data refer, numbered according to the number sequence used in the original MultiPic dataset^21^. The column **Number of Responses** corresponds to the number of individual responses collected for each item in each language (namely, the number of participants who provided an answer). The column named **H Statistic** includes the level of agreement in the responses for a given item in a given language across participants as measured by the H index^29^, which increases as a function of response divergence. The column **Modal Response** includes the strings corresponding to the most frequent response for each item in each language; note that in cases in which the same level of agreement was found for two different responses, both are presented separated by a “/” symbol (e.g., response1/response2). The column labelled **Modal Response Percentage** corresponds to the percentage of responses corresponding to the modal response out of all valid responses (namely, responses for each item in each language that do not correspond to “I don’t know” or idiosyncratic responses). The column **“I don’t know” Response Percentage** provides the percentage of participants in each language who did not know the name of the displayed element and selected the corresponding button. The **Idiosyncratic Response Percentage** column includes the percentage of responses to each item in each language that were provided only by a single participant (*N* = 1). Finally, the column labelled **Familiarity** includes the mean familiarity score calculated from the total responses to each item using the 0-to-100 scale of all participants in each language or language variety. Supplementary Table presents a summary of the descriptive statistics of these measures for each language or variety, with the only exception being familiarity measures for those included in the original study^21^, since their items were not normed for this factor.

## Technical Validation

**Fig. 1:**
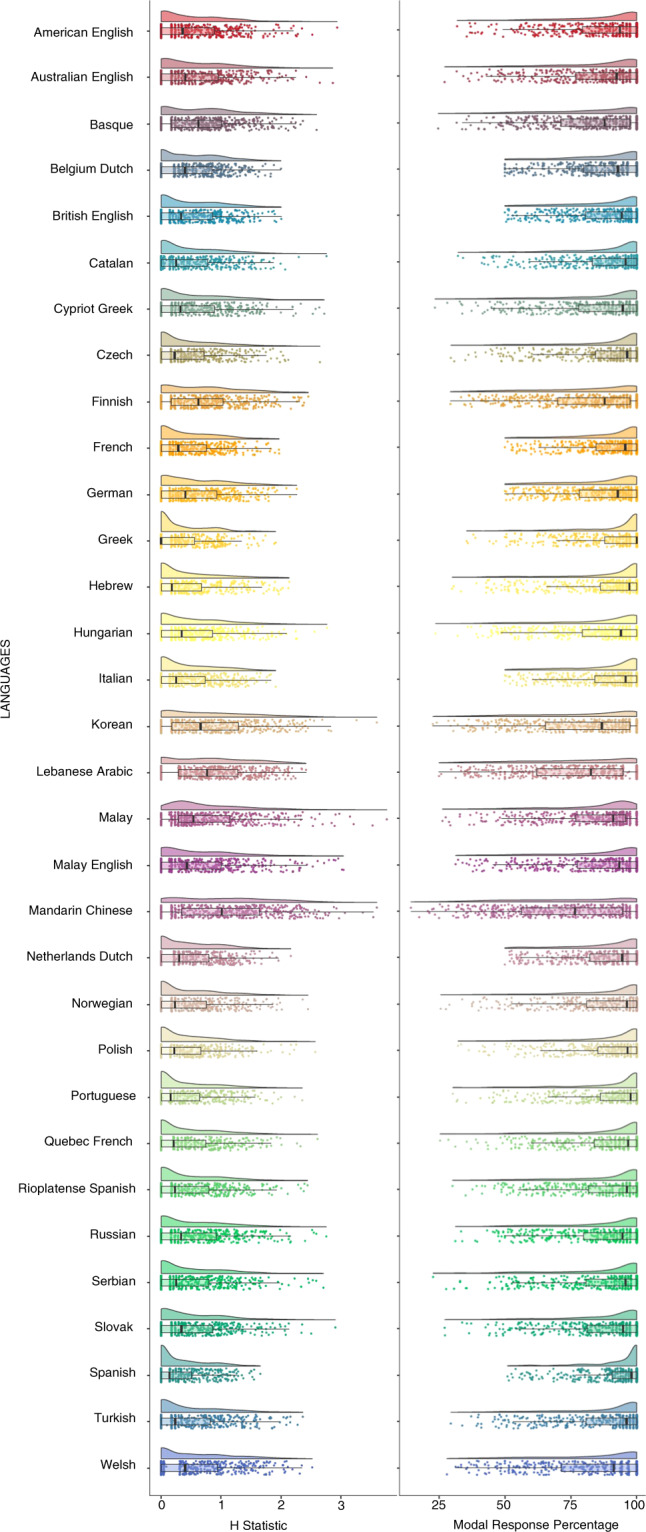
Density plots of the H Statistic and the Modal Response Percentage across items in each of the languages and varieties. Dots represent mean values for individual items and vertical black lines represent mean values across items.

First, a descriptive analysis was performed to validate that the resulting datasets per language or variety were of sufficient quality. To this end, two measures were analysed across languages or varieties: the mean H statistic and the mean modal response percentage. All analyses were done using Jamovi^30^ and R^31^. The mean H statistic of the current general dataset was of 0.53 (standard deviation = 0.58), with values ranging between the lower bound of 0.30 (Spanish) and the upper limit of 1.07 (Mandarin Chinese). The mean value of the H statistic is in line with those reported in earlier normative studies with different materials (e.g., 0.67 in^17^; 0.55 in^18^; 0.68 in^9^; 0.32 in^13^), and not surprisingly, aligns with the mean H statistic of 0.74 reported for the general set of 750 drawings normed in^21^. (Note in this regard that stimuli selection for the current study considered 500 items with the highest name agreement from the original study in the 6 languages or varieties tested). The mean modal response percentage of the general dataset was 86.8% (standard deviation = 16.5). The language with a lower percentage of modal response is Mandarin Chinese (73.30%), and the language with a higher percentage is Spanish (93%). These values are similar to the 80% reported in the original study^21^, and closely approach the mean modal response percentages provided in earlier studies with different sets of stimuli (e.g., 85% in^8^; 87% in^18^; 87% in^3^). Together, the relatively low mean H statistics and the high mean modal response percentages of the current dataset suggest a high name agreement across items, languages and varieties, validating the materials for their use in different kinds of experiments and tests. Fig. 1 illustrates the density plots of the H Statistic and the Modal Response Percentage in each language/language variety.

Second, a series of correlation analyses were conducted to validate individual dataset quality. To that end, and considering that there is no a priori reason to expect cross-language similarities in name agreement measures, since each language has its own particular lexicon, initial focus was on familiarity values. While the specific name or names used to refer to an entity can easily vary across languages, yielding heterogeneous name agreement scores, the way the materials were created and selected pointed to high familiarity with the entities depicted across cultures. Consequently, reasonably high cross-language correlation coefficients were expected between familiarity scores. A correlation analysis performed on the different familiarity scores obtained for each item in each language showed that all the Pearson pairwise correlation coefficients were significant at the p < 0.001 level, with r-values ranging between 0.351 (Catalan vs. Turkish) and 0.919 (Greek vs. Cypriot Greek), and a very high mean correlation coefficient of 0.702 across tests.

As a final validation analysis, we took a close look at the pool of varieties from the same language, since it was expected that results for different dialectal forms or varieties of a given language would elicit similar responses across measures. To this end, the name agreement in the 4 different varieties of English that were included in the dataset (i.e., American English, Australian English, British English, and Malaysian English) were analysed. A correlation analysis of the H statistic showed that responses overlapped highly across varieties, with the lower r-value being 0.579 (American English vs. Malaysian English) and the highest being 0.772 (American English vs. Australian English), and all correlations being significant at the p < 0.001 level. Similarly, the mean percentage of modal responses was also significantly correlated across varieties, with r-values ranging between 0.551 (American English vs. Malaysian English) and 0.759 (American English vs. Australian English), again with all p-values being below 0.001.

## Supplementary information

Supplementary Table

## Data Availability

No custom code was used to generate or process the data described in the manuscript.
