# Supplementary material for: The Multilingual Picture Database
Source: Sci Data. 2022 Jul 21;9:431. doi: 10.1038/s41597-022-01552-7 (PMC9304413; doi:10.1038/s41597-022-01552-7)
Supplement: Supplementary file 1 — Supplementary Table [file 41597_2022_1552_MOESM1_ESM.docx]

**Supplementary Table.** Summary data of the whole set of responses per language or language variety. Mean values are provided with standard deviations in parenthesis.

| **Language** | **Number of**  **Participants (females)** | **Mean age (SD)** | **H**  **Statistic** | **Modal**  **Response**  **Percentage** | **"I don't know"**  **Response**  **Percentage** | **Idiosyncratic**  **Response**  **Percentage** | **Familiarity** |
| --- | --- | --- | --- | --- | --- | --- | --- |
| American English | 80 (57) | 22.2 (9.4) | 0.53 (0.55) | 87.45 (15.34) | 0.00 (0.00) | 3.29 (3.18) | 84.31 (6.05) |
| Australian English | 80 (40) | 21.8 (4.2) | 0.57 (0.57) | 85.81 (16.41) | 1.03 (1.98) | 2.80 (3.17) | 80.64 (6.71) |
| Basque | 80 (42) | 26.7 (10.9) | 0.66 (0.58) | 82.94 (17.24) | 2.34 (4.28) | 1.93 (2.19) | 74.56 (7.69) |
| Belgium Dutch | 60 (43) | 22.8 (4.0) | 0.5 (0.48) | 87.59 (13.88) | 2.16 (3.93) | 2.89 (3.16) | - |
| British English | 100 (71) | 19.5 (3.5) | 0.48 (0.5) | 88.53 (13.62) | 2.89 (5.83) | 1.55 (1.69) | - |
| Catalan | 98 (51) | 23 (6.2) | 0.45 (0.51) | 88.98 (14.56) | 1.70 (3.30) | 1.12 (1.44) | 75.46 (8.84) |
| Cypriot Greek | 80 (52) | 23.6 (5.1) | 0.52 (0.57) | 86.69 (16.84) | 1.95 (3.59) | 3.12 (2.46) | 80.66 (8.25) |
| Czech | 83 (43) | 24.9 (4.3) | 0.42 (0.51) | 89.33 (15.40) | 0.52 (1.80) | 1.65 (2.22) | 83.61 (7.78) |
| Finnish | 84 (21) | 25.6 (4.39) | 0.69 (0.61) | 82.14 (18.14) | 1.05 (2.78) | 3.03 (2.84) | 82.30 (6.98) |
| French | 100 (88) | 20.2 (2.8) | 0.44 (0.47) | 90.19 (12.26) | 1.61 (4.09) | 2.00 (2.15) | - |
| German | 100 (60) | 22.2 (3.1) | 0.55 (0.52) | 87.33 (13.90) | 0.81 (2.72) | 2.35 (2.41) | - |
| Greek | 80 (72) | 23.3 (5.7) | 0.3 (0.42) | 91.43 (13.69) | 0.78 (2.19) | 1.62 (2.49) | 80.69 (10.57) |
| Hebrew | 80 (63) | 23.1 (4) | 0.4 (0.51) | 89.61 (15.60) | 1.51 (3.88) | 1.56 (2.22) | 88.82 (6.97) |
| Hungarian | 78 (38) | 25.19 (5.31) | 0.51 (0.55) | 86.77 (16.58) | 0.87 (2.68) | 1.96 (2.41) | 88.86 (4.90) |
| Italian | 100 (68) | 24.5 (5.7) | 0.43 (0.47) | 89.76 (13.17) | 2.00 (3.87) | 1.37 (1.62) | - |
| Korean | 78 (42) | 23.6 (2.8) | 0.8 (0.72) | 79.96 (20.02) | 2.35 (4.98) | 4.36 (4.26) | 69.61 (9.58) |
| Lebanese Arabic | 40 (29) | 26.6 (5.4) | 0.8 (0.65) | 77.79 (20.36) | 9.91 (11.02) | 7.67 (7.27) | 81.63 (9.95) |
| Malay | 80 (61) | 20.6 (2.6) | 0.78 (0.67) | 84.26 (16.38) | 3.84 (7.46) | 2.14 (2.68) | 80.67 (8.06) |
| Malaysian English | 80 (63) | 21.6 (3.1) | 0.64 (0.67) | 85.84 (17.33) | 2.31 (4.06) | 3.54 (2.91) | 84.91 (5.81) |
| Mandarin Chinese | 80 (62) | 20.7 (2.5) | 1.07 (0.79) | 73.29 (21.92) | 1.89 (4.71) | 7.60 (5.91) | 74.24 (6.54) |
| Netherlands Dutch | 60 (42) | 21.1 (2.4) | 0.45 (0.48) | 88.80 (13.54) | 1.29 (3.66) | 2.68 (2.80) | - |
| Norwegian | 80 (57) | 30.8 (12.5) | 0.43 (0.52) | 88.30 (15.93) | 0.78 (2.43) | 2.08 (2.47) | 79.73 (7.88) |
| Polish | 89 (45) | 20.1 (1.5) | 0.4 (0.49) | 89.89 (14.34) | 1.06 (3.07) | 1.70 (2.04) | 87.10 (5.20) |
| Portuguese | 88 (81) | 21.38 (4.19) | 0.37 (0.48) | 90.38 (14.72) | 1.67 (3.90) | 1.38 (1.71) | 77.16 (10.23) |
| Quebec French | 63 (44) | 23.0 (3.55) | 0.42 (0.52) | 89.00 (15.66) | 0.83 (2.82) | 1.63 (2.27) | 85.21 (8.47) |
| Rioplatense Spanish | 79 (62) | 36,65 (12.2) | 0.45 (0.54) | 88.72 (15.44) | 0.78 (2.31) | 1.55 (1.72) | 82.23 (8.64) |
| Russian | 82 (41) | 25.63 (7.21) | 0.53 (0.59) | 87.20 (16.04) | 0.75 (2.38) | 1.78 (2.19) | 86.24 (6.21) |
| Serbian | 95 (58) | 22.31 (5.01) | 0.47 (0.56) | 87.97 (16.45) | 0.81 (2.47) | 1.41 (1.93) | 87.44 (5.89) |
| Slovak | 80 (61) | 26.7 (8.63) | 0.5 (0.54) | 87.76 (15.57) | 0.93 (3.04) | 2.99 (2.36) | 86.78 (6.92) |
| Spanish | 100 (61) | 23.9 (4.3) | 0.3 (0.4) | 92.95 (11.06) | 0.98 (2.65) | 1.09 (1.47) | - |
| Turkish | 80 (40) | 29.6 (9.0) | 0.46 (0.54) | 87.86 (16.16) | 1.14 (3.36) | 2.01 (2.51) | 90.95 (6.04) |
| Welsh | 36 (32) | 24.51 (8.91) | 0.55 (0.6) | 83.87 (18.82) | 4.97 (7.38) | 2.57 (3.46) | 78.52 (9.03) |
